# A Granite Bed Storage for a Small Solar Dryer

**DOI:** 10.3390/ma11101969

**Published:** 2018-10-13

**Authors:** Magdalena Nemś, Artur Nemś, Paweł Pacyga

**Affiliations:** Faculty of Mechanical and Power Engineering, Wroclaw University of Science and Technology, Wybrzeże Wyspiańskiego 27, 50-370 Wroclaw, Poland; artur.nems@pwr.edu.pl (A.N.); pawel.pacyga@pwr.edu.pl (P.P.)

**Keywords:** granite spheres, heat storage, solar dryer

## Abstract

The article presents the concept of a high-temperature solar dryer with an internal bed storage. Granite was selected as the material for filling the bed storage, and an emphasis was put on its versatile use and favourable thermal and mechanical properties. Experimental tests were carried out for the charging process of the bed storage, which was filled with granite spheres with three different diameters. The influence of the sphere’s diameter on the charging and discharging process of the bed storage was analysed. The results of the experiment allowed a conclusion to be drawn that the use of a granite storage bed could prolong the operation of the considered dryer by two hours.

## 1. Introduction

The process of drying food for its preservation is very common. In most dryers, heat from fossil fuel combustion or electricity is used for the drying. The popularity of dryers that use solar radiation is still growing. There are numerous literature reviews [1,2], as well as research concerning only solar food drying [3,4]. However, there is a problem with the variable availability of solar radiation during the day, and the number of hours of sunshine. Sometimes, it is not enough to ensure the proper parameters of the drying process. The solution to this problem is the use of a bed storage inside a solar dryer. In the case of temporary cloudiness, the accumulative material will provide adequate conditions to the end of the drying process for a specified period of time, which depends on the used solution.

## 2. Materials and Methods

Solar dryers are characterised by their simple construction. Most often, they do not use a heat exchanger or intermediate medium, and the working medium that flows through the accumulative material is directed towards the products being dried. Therefore, the choice of material for a bed storage is important, especially with regards to the safety of drying food. The selected material cannot release any toxic compounds or impurities over time, and during contact with the hot working medium. It should also be durable in order to reduce the need for servicing and system downtime.

In solar drying installations, accumulation techniques that use both sensible and latent heat are used. Extensive reviews on this topic can be found in [5,6,7]. Currently, phase-change materials are very popular, among which the use of paraffin wax dominates, e.g., for drying red chillies [8] or mushrooms [9]. The current research also concerns a dryer with phase-change material, which is dedicated to medicinal herbs [10] and crops [11]. The main problem with the use of phase-change materials is that it is necessary to guarantee the appropriate temperature ranges of the process during charging and discharging. This can be a difficult condition to meet in simple solar dryers due to the variable availability of solar radiation.

Solid phase materials can be found, among others, in solar dryers with an oil tank [12], a battery made of concrete, as well as sand and rock bed [13]. The use of rocks as a material for filling a bed storage enables the construction of a simple drying installation that uses air as the working medium. Gravel [14] and granite grits [15,16,17] are also frequently seen as materials that can be used for filling bed storages in food dryers.

### 2.1. Granite

Granite is an intrusive rock that is formed at a great depth below the Earth’s surface as a result of volcanic magma being exposed to high pressure and hardening. This type of rock can be found in many regions of the world. Significant amounts of granite are extracted in the United States (USA), China, Africa, Scandinavia, and India. In Poland, granite deposits are found in Lower Silesia and the Tatra Mountains [18]. The resources of stone materials in Poland amount to 8415 million tons, 48% of which are made of igneous rock with an estimated extraction of 51% [19]. A lot of information about granite regarding its structural and geological problems can be found in [20].

Research on granite has been going on for years, which is why it is a well-known material. In most cases, this relates to the possibilities of its application. The basic area for the use of granite is the construction industry. In [21], authors investigated the potential use of locally available waste materials from a limestone quarry and the granite industry as a partial replacement for cement. In other studies [22], the use of polarising petrographic and fluorescence microscopic techniques to study the development of microcracks was proposed.

The technique proved to be very useful when choosing granites that are suitable to be used as building materials in environments and climates that are characterised by the occurrence of thermal stresses. Granite is also used to protect the slopes of mountains, riverbanks, and underground constructions. This is why knowledge about its mechanical properties is very important. In [23], an extensive literature search was performed in order to compile a database of laboratory compression testing data for granitic rock. In [24], experimental laboratory tests and numerical simulations of a grain-based approach in two-dimensional particle flow code (PFC) were conducted on the mechanical strength and failure behavior of local granite.

In [25], the bending properties of granite beams were investigated. A large group of articles is related to research on granite with regards to its use for the production of geothermal energy. The heat transfer characteristics between flowing water and a granite fracture wall [26] are determined, the effect of heating and cooling on the material is investigated [27], and the numerical models of a granite reservoir are created [28]. Granite can also be used as a filling material for bed storages that cooperate with a high temperature solar installation in both a solar plant [29] and a residential building [30]. The storing of radioactive materials in granite [31,32] is another one of its interesting applications. The cited examples demonstrate the interesting properties and wide range of use of granite.

The following properties of granite distinguish it from other standard materials:

- heat storage—stone absorbs heat well, stores it for a long time, and most importantly releases it at an even rate. For this reason, it is frequently used as a component of packed beds. It is worth mentioning that granite must not be subjected to rapid changes of temperatures, as this may cause it to break.
- temperature resistance—up to 1000 °C.
- hardness—depending on the quartz content, this type of stone has a number of six or seven in the 1–10 Mohs scale of mineral hardness, which means that it is harder than steel.
- density—granite has a high density: approximately 2700 kg/m^3^ [19].
- safe when in contact with food (used both in dryers and solar cookers).
- durable even with many cycles and use in high temperatures [29].

**Figure 1:**
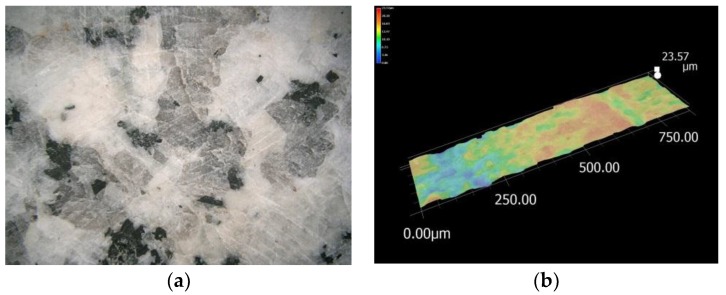
Grinded Strzegom granite; (**a**) photo of the surface, (**b**) roughness.

After the completion of the literature review, it was decided to test granite as a filling material for a packed bed in a solar dryer. The locally available Strzegom granite has high technical properties (compressive strength of 110–170 MPa [19]) and high durability. Figure 1 shows a photo of the surface of the storage material and a microscope photo on which surface roughness is visible. It is equal to 24 μm for a grinded surface. For comparison, the roughness of rough granite elements, such as breakstone, can reach up to 276 μm.

## 3. The Concept of a Solar Dryer with Internal Heat Storage

**Figure 2:**
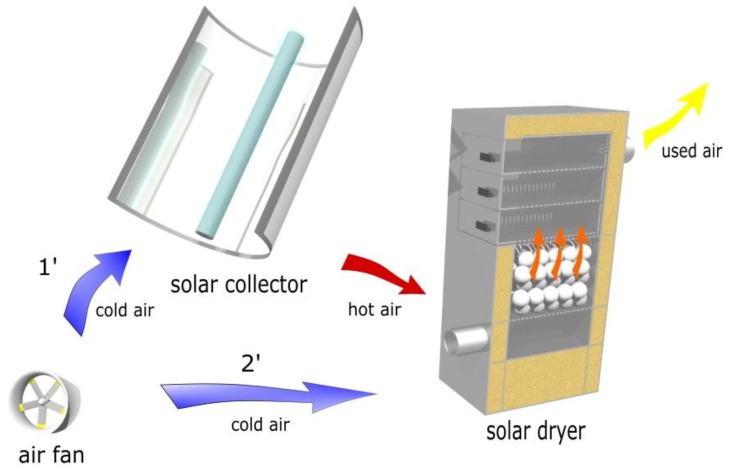
Visualisation of a solar dryer with a granite bed storage for two cases; 1’ when the heat comes from the collector, and 2’ when the heat comes from the bed discharge process.

The proposed technological solution is shown in Figure 2. The main elements of the system are a concentrating collector that cooperates with a dryer that has internal heat storage. The authors verified that the use of a solar collector in Polish climatic conditions enables high temperatures to be obtained not only in summer, but throughout the year [30]. The air heated in the collector is directed through the filling material toward the material that is being dried (operating mode marked in Figure 2 as 1’). During this work, the material is dried at the same time as the charging of the rock bed storage. After sunset or when there is sudden cloud cover and no heat generation by the solar collector, the air is directed to the dryer, bypassing the collector. There is then an inflow of cold air at ambient temperature to the dryer. The air flowing through the granite filling is heated and directed to the material being dried (the operating mode is indicated in Figure 2 as 2’). During this time, there is a process of discharging the bed storage.

The considered dryer can be used for both a low and high temperature-drying process due to the use of a solar concentrating collector. The proposed technical solution allows the hot medium from the collector to be mixed with fresh air taken from the surroundings. A solar air heater operating in Polish climatic conditions was the subject of research conducted by one of the authors [33,34]. In order to intensify the heating rate of the flowing air, the absorber was equipped with internal ribs. As a result of theoretical research of such a solar collector [33], it was possible to determine the optimal range of air volume flow rate from 0.005 m^3^/s to 0.01 m^3^/s at an efficiency of about 40%. In turn, experimental tests indicated the range of air outlet temperatures to be from approximately 80–160 °C for the above flow rates [34]. These parameters were taken into account when creating the test plan for the granite bed storage for a small solar dryer.

A high temperature of the working medium of up to 150 °C [35] is necessary, among others, in a two-stage drying process, e.g., for cereals or corn. Other high-temperature drying processes involve the maximum allowable temperature of 150 °C for cassava, 140 °C for tea, and 90 °C for chillies [5].

## 4. Experimental Test Set-up

**Figure 3:**
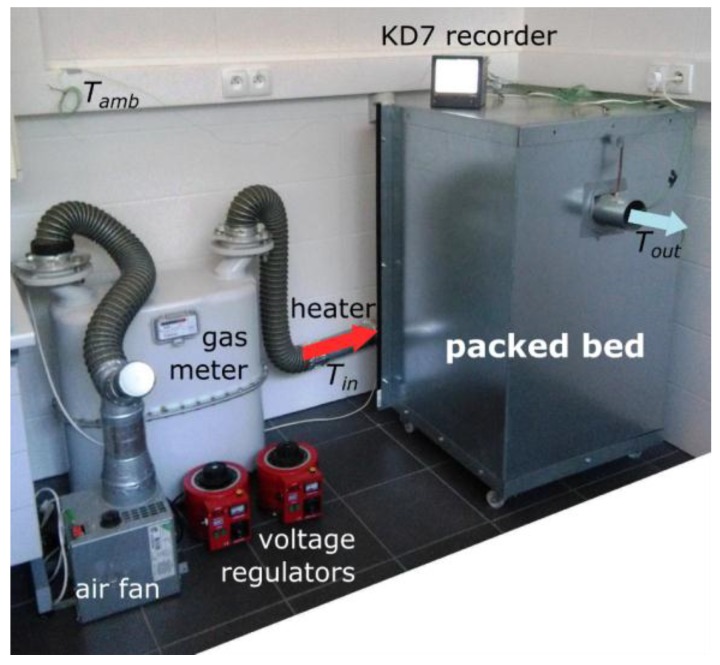
Experimental set-up.

The experimental test set-up (Figure 3) is located in a laboratory of the Department of Fundamentals of Construction and Flow Machines at Wroclaw University of Science and Technology, Poland. Previous research with the use of a test stand that included the model of heat exchange for a ceramic filled bed storage is presented in [36]. The authors described research concerning crushed granite in [30], which included the analysis of heat exchange during the operation of a bed storage, and allowed the temperature of the filling material for the long-term accumulation system to be determined. The main purpose of the current work is to choose the appropriate diameter of filling material for a rock bed storage that is integrated with a dryer. The bed storage is meant to stabilize the system by heating the air directed to the products being dried during a period of low solar radiation. 

The experimental stand consists of the following elements:

- a fan providing air flow through the rock bed;
- a gas meter for measuring the volume flow rate of air injected into the rock bed;
- heaters for regulating the temperature of the injected air;
- a packed bed thermal energy storage;
- autotransformers: for both the temperature and the air fan;
- a voltage regulator;
- a thermocouple set with a KD7 recorder;
- rock bed filler material comprising of granite spheres.

After the test stand is activated, the air fan forces the air stream into the packed bed. The stream first passes through the gas meter, which enables its air volume flow rate to be regulated. In the next step, the air stream is heated to a predefined temperature with the heater, and then it flows through the packed bed filled with spheres of various sizes. The air flow and the air temperature are regulated with the two autotransformers.

## 5. Efficiency of the Charging Process

The performed tests allowed the actual and the maximum amount of accumulated energy to be calculated, and as a result, the efficiency of the charging process to be determined [37].

The efficiency of the packed bed charging process is described in Equation (1):

(1) $$\;\varepsilon =\;\frac{{\dot{Q}}_{act}}{{\dot{Q}}_{max}}\;$$

The actual flux of accumulated energy is described in Equation (2), and is calculated from the balance of energy at the input to the system and the heat losses on the surface of the packed bed:

(2) $$\;{\dot{Q}}_{act}=\;{\dot{Q}}_{max}-{\dot{Q}}_{loss},\;\mathrm{kW}\;$$

Equation (3) describes the maximum flux of accumulated energy, which is related to the parameters of the working medium treated as perfect gas at 20 °C ($\rho_{a}$ = 1.2 kg/m^3^,$\;c_{pa}$ = 1.005 kJ/kg·K), because in the analysed temperature range of 20–150 °C, the specific heat of air at a constant pressure only changes by 1.2%. The density was only used for calculating the mass flow rate, and during the tests, the volumetric flow rate of the air sucked from the surroundings was measured, according to Equation (3):

(3) $$\;{\dot{Q}}_{max}=\;{\dot{m}}_{a}\times c_{pa}\times \Delta T=\;{\dot{V}}_{a}\times \rho_{a}\times c_{pa}\times \left(T_{in}-T_{out}\right),\;\mathrm{kW}\;$$

The flux of heat loss from the storage bed (4) is calculated in the exact same way as the maximum flux of accumulated energy, but for a storage bed that is not filled with granite:

(4) $$\;{\dot{Q}}_{loss}=\;{\dot{m}}_{a}\times c_{pa}\times \Delta T_{loss}=\;{\dot{V}}_{a}\times \rho_{a}\times c_{pa}\times \left(T_{in.loss}-T_{out.loss}\right),\;\mathrm{kW}\;$$

The following factors influence the efficiency of the packed bed charging process:

- the type of packed bed filler material;
- the geometry of the packed bed;
- the type and thickness of thermal insulation used;
- the shape and size of filler material;
- the air inlet temperature;
- the volume flow rate of air injected into the bed;
- the ambient temperature;
- the bed charging time.

The majority of the above-mentioned factors varied during the experiment, which was natural for the assumed packed bed design, and the ambient temperature was approximately 19 °C. Thus, three variables were considered as independent variables: $x_{1}=T_{in}$, $x_{2}=$ *D*, and$\;x_{3}={\dot{V}}_{p}$. In its general form, the theoretical charging process efficiency is a function of these three independent variables (5):

(5) $$\varepsilon_{t}\;\times \;(T_{in},\times \;D,\times \;{\dot{V}}_{a})$$

### 5.1. Hartley Experiment Design for the Investigated Packed Bed

Of the three-level experiment designs, it was decided that the tests that aimed to determine the packed bed’s charging characteristics would be based on the Hartley design [38]. It is a very convenient design that allows the time and the cost of the experiment to be reduced due to the three input factors positioned on three variability levels only requiring 11 measurement sessions instead of 3^3^, i.e., 27 required sessions in the case of other experiment designs. The input variables included: temperature of the inlet air, air flow stream, and rock bed filler material—granite spheres of various sizes. The temperatures were measured at an interval of one minute, and recorded on an external device.

Table 1 lists the assumed values of the input factors, and Table 2 shows the matrix of the Hartley design for the performed experiment.

The Hartley design serves as a means to generate a quadratic equation describing the unknown parameter with the use of three independent variables. In the analysed case, a series of experiments that were performed in accordance with the design allow the following polynomial to be obtained (6):

(6) $$\;\varepsilon_{t}=\;b_{o}+b_{1}\times T_{in}+b_{2}\times T_{in}^{2}+b_{3}\times D+b_{4}\times D^{2}+b_{5}\times {\dot{V}}_{a}+b_{6}\times {\dot{V}}_{a}^{2}+b_{7}\times T_{in}\times D+\;b_{8}\times T_{in}\times {\dot{V}}_{a}+b_{9}\times D\times {\dot{V}}_{a}+b_{10}\times T_{in}\times D\times {\dot{V}}_{a}\;$$

Then, 11 unknown regression coefficients are calculated from relationship (7), which corresponds to the results of the matrix analysis:

(7) $$\;b_{i}=\;\frac{Matrix\;determinant\;i}{Normal\;matrix\;determinant}\;$$

The matrix determinant is a real number assigned to a square matrix. Let us consider a given square matrix *A* of order *n*. The determinant is such a representation that assigns exactly one real number *detA* to a given matrix. If the matrix is of the *n* = 1 order, its determinant is *detA* = *a*_11_. If the matrix order is greater than 1, its determinant is calculated according to Equation (8):

(8) $$\;detA=\;\overset{n}{\underset{i-1}{\sum }}{\left(-1\right)}^{i+j}a_{ij}detM_{ij}\;$$

where $detM_{ij}$ is the determinant of the matrix obtained from matrix *A* by deleting the *i*th row and the *j*th column [39].

**Figure 4:**
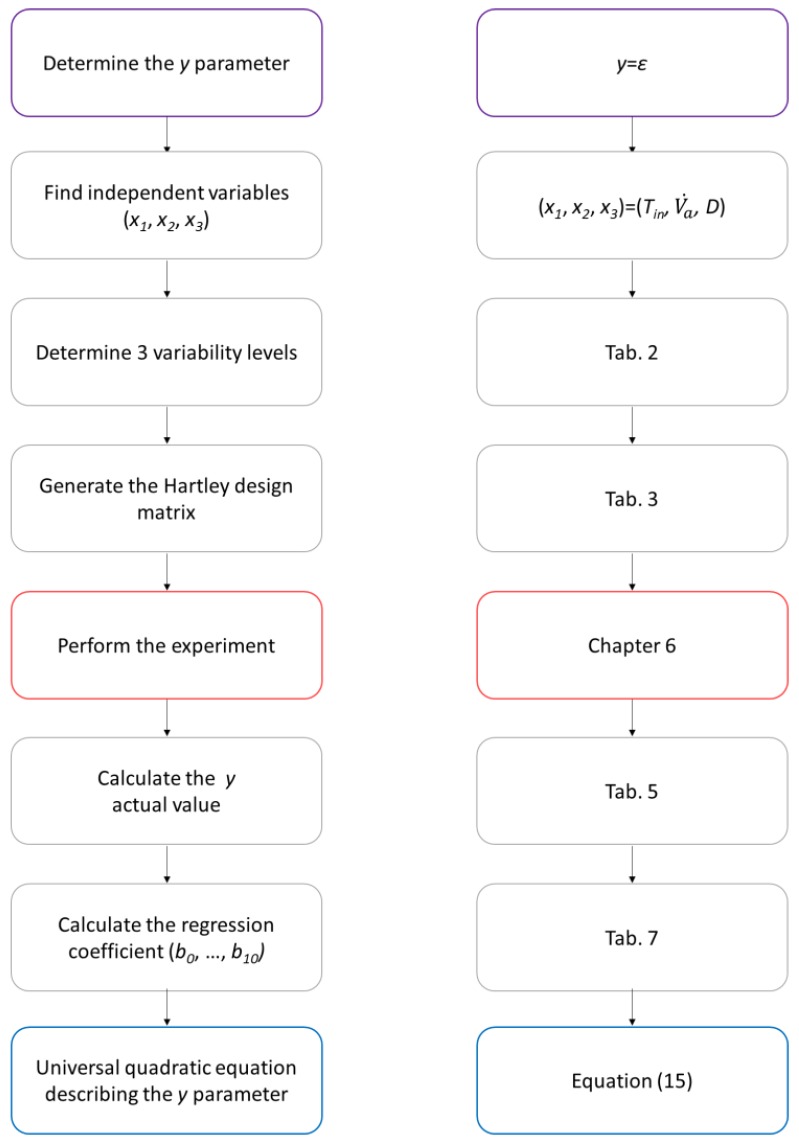
Algorithm of the Hartley design procedure.

Figure 4 shows the subsequent steps of the Hartley design and their corresponding locations in this paper, which are provided in order to clarify the procedure’s algorithm.

## 6. Experimental Tests for Rock Bed Storage

**Figure 5:**
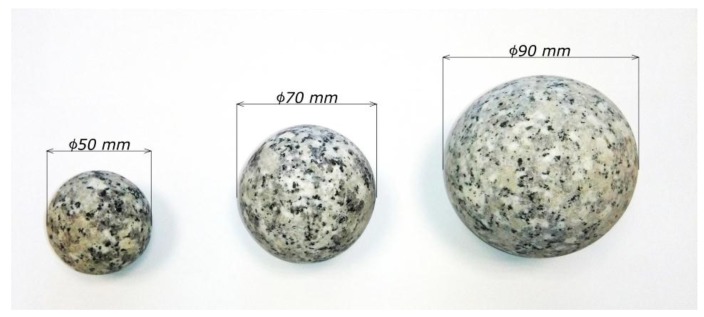
Granite spheres used in the experiment.

Eleven series of tests were performed on the experimental set-up in accordance with the Hartley design of the experiment for three different variability levels: air temperature at the inlet to the packed bed, air volume flow rate, and three various diameters of the granite spheres. The number of granite spheres in the packed bed was dependent on their mass: for each of the three diameters, the total mass of spheres was 25.6 kg. The temperature was measured with thermocouples, which were positioned at the inlet of the packed bed, at the outlet of the packed bed, and also outside the experimental set-up, in order to determine the ambient temperature. The time of each of the experiments exceeded three hours, and lasted until the temperature of the air at the inlet to the packed bed stabilized. The spheres that were used in the experiment are shown in Figure 5.

The material, in the shape of spheres, was selected for the tests since in the calculations of heat exchange in rock bed storages, the equivalent diameter is determined and the material is treated as a sphere [40]. This allowed any error related to determining the equivalent diameter to be avoided, which is troublesome.

**Figure 6:**
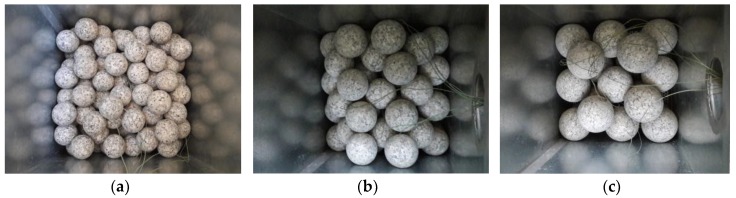
Spheres of (**a**) 50 mm, (**b**) 70 mm, and (**c**) 90 mm in diameter arranged in the packed bed.

Figure 6 shows spheres of various sizes arranged in the packed bed. The bed was filled in configurations with either 26 large spheres, 58 medium-size spheres, or 142 small spheres.

In order to determine the heat loss flux for each of the configurations, an additional measurement series were performed for a configuration without granite filling. The investigations covered the mass air flow rate, as well as the air inlet and outlet temperatures. In the next step, second-degree polynomials were determined for the relation between the temperature decrease and temperature difference for each of the three investigated air flow rates. By inserting the resulting temperature decrease values into Equation (4), it was possible to find the heat loss flux from the packed bed.

Table 3 contains the measurement results, as well as the mean temperature and the temperature difference.

**Figure 7:**
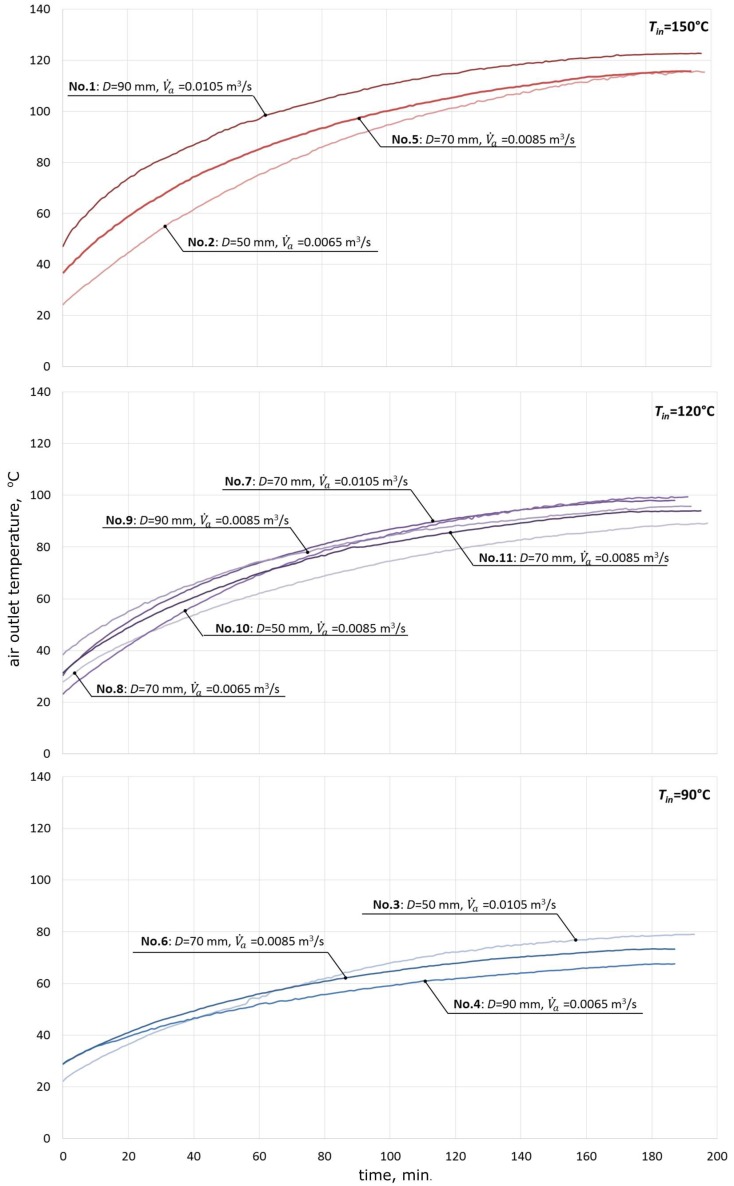
The relationship between the air temperature at the outlet of the packed bed and the time for 11 measurement series.

Using the plan of the experiment and data from Table 2, the temperature values were determined. They enabled the dependence between air outlet temperatures over time to be determined, as shown in Figure 7.

The generated relationships between the air temperatures at the outlet of the packed bed and time allowed the observation that an increase of the temperature at the inlet to the bed is accompanied by an increased difference between the temperatures of air at the inlet to the bed and at the outlet of it. For instance, with no consideration given to the size of the granite spheres, at the air inlet temperature of 150 °C, the mean difference is 32.0 °C, at the air inlet temperature of 120 °C, the mean difference is reduced to 24.7 °C, and at the lowest inlet temperature, the difference is only 16.7 °C.

Table 4 contains the results of calculations for 11 measurement series. The efficiency of the packed bed charging process and its constituent elements were calculated in accordance with equations (1–4). In each of the series, the measurements were finished when the increase of the air outlet temperature did not exceed 1 °C over a 10-minute charging process time.

**Figure 8:**
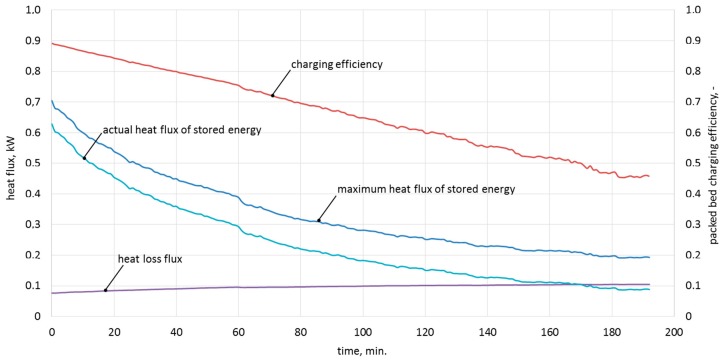
Characteristics of the maximum and actual heat flux, heat loss, and charging efficiency in the function of time for experiment No. 9.

In order to clarify the experimental procedure, Table 5 shows the measured values and calculations for 10-minute time steps in the ninth measurement series, while Figure 8 represents the characteristics of the maximum, actual heat flux, and efficiency of the charging process in the function of time for experiment No. 9.

As was shown in Figure 8, the process efficiency decreases over time. This is due to the decreasing flux of transferred heat, which after three hours is smaller than the losses from the bed storage. Further charging of the bed storage is a low-efficiency process.

### 6.1. Granite Bed-Charging Process Characteristics

Based on the matrix determinants, it was possible to obtain the values of regression coefficients and collect them in Table 6. A general quadratic equation was established for calculating the theoretical efficiency of the packed bed charging process $\varepsilon_{t}$. The calculations of the theoretical charging process efficiency are based on the values of regression coefficients $b_{i}$.

The general quadratic Equation (6) for calculating the theoretical efficiency of the packed bed charging process, after one hour, takes the following form (9):

(9) $$\;\varepsilon_{t}=-0.26422332+0.00722428567\times T_{in}\;+\;-0.00000739681743\times T_{in}^{2}+11.9773576\times D-24.039309\times D^{2}+103.480696\times {\dot{V}}_{a}+\;+344.957618\times {\dot{V}}_{a}^{2}-0.0516405955\times T_{in}\cdot D-0.670653395\times T_{in}\times {\dot{V}}_{a}+1176.47086\times D\times {\dot{V}}_{a}++6.66838861\times T_{in}\times D\times {\dot{V}}_{a}\;$$

**Figure 9:**
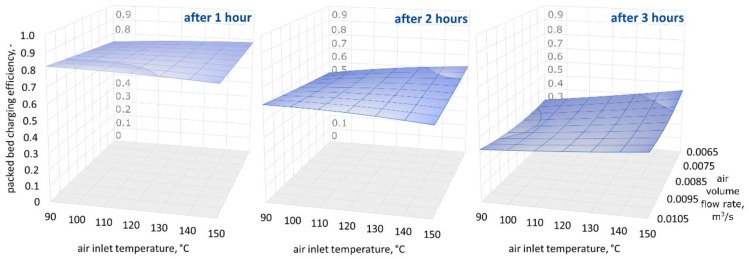
Relationship between the air inlet temperature, the air volume flow rate, and the theoretical efficiency of the packed bed charging process, for a granite sphere diameter equal to 50 mm, after one, two, and three hours of the charging process.

**Figure 10:**
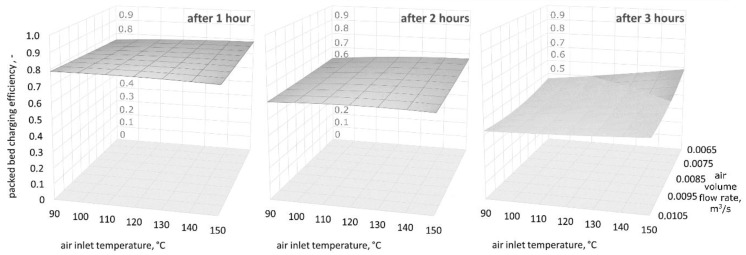
Relationship between the air inlet temperature, the air volume flow rate, and the theoretical efficiency of the packed bed charging process, for a granite sphere diameter equal to 70 mm, after one, two, and three hours of the charging process.

**Figure 11:**
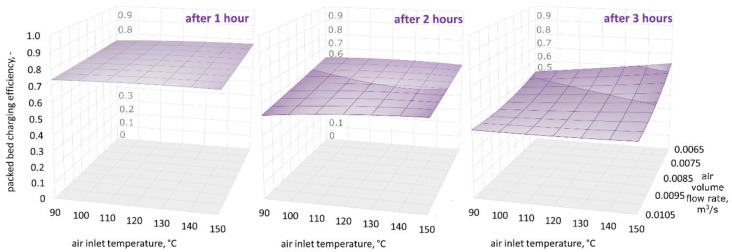
Relationship between the air inlet temperature, the air volume flow rate, and the theoretical efficiency of the packed bed charging process, for a granite sphere diameter equal to 90 mm, after one, two, and three hours of the charging process.

Figure 9, Figure 10 and Figure 11 show three-dimensional (3D) graphs for the following variables: air inlet temperature *T_in_*, air volume flow rate ${\dot{V}}_{a}$, and theoretical efficiency of the packed bed charging process $\varepsilon_{t}$, which were plotted for constant granite sphere diameter values *D*, equal to 50 mm, 70 mm, and 90 mm, respectively.

Figure 9, Figure 10 and Figure 11 demonstrate that the highest values of the packed bed’s theoretical charging process efficiency are observed at the beginning of the accumulation process and decrease with the bed charging time. The efficiency variations in the functions of air inlet temperature and volume air flow rate show a similar pattern for all of the sphere diameters after one, two, and three hours of charging time. Remarkably, after one hour of charging time the highest efficiency values are observed for spheres with the smallest diameter. After two hours of charging time, the efficiency is highest for the 70-mm spheres, and after three hours, the efficiency is highest for the largest spheres. This phenomenon may be caused by the smallest spheres having a greater heat exchange surface. As a result, in the initial phase of the process, the smallest spheres show the highest temperature increase, but the flux of accumulated heat is decreasing in the latter phases. It is worth mentioning the two investigated configurations in which the charging process efficiency is high for all three diameters: at the beginning of the accumulation process for the smallest air flow rates and the low inlet temperature of the medium, and after three hours for the greatest air flow rates and the highest air inlet temperature values.

### 6.2. Granite Bed Discharging Process

**Figure 12:**
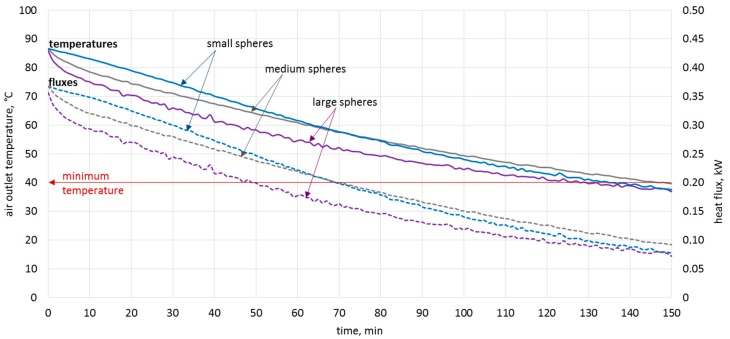
Discharging of rock bed filled with spheres of various diameters.

Due to the use of the bed storage in the dryer, it is possible to continue the process despite temporary cloud cover. Figure 12 shows the process of discharging the bed storage for different sizes of filling. The bed storage was heated to 90 °C. This corresponds, for example, to the maximum allowable drying temperature of chillies [5]. A temperature of 40 °C [41] was assumed as the limit temperature, below which the continuation of the drying process of chillies has no technological and economic sense. In all three cases, the drying process could take place for more than two more hours, despite the lack of solar radiation. 

In addition to the temperature, the heat flux during the discharging of the bed storage was analysed. The flux values are similar, but the highest heat flux during two hours of discharge was achieved for the spheres with the smallest diameter. However, the smallest heat flux was obtained from the largest spheres. This means that for this filling, the process is slower, and can therefore sustain the drying process longer without the flow rate needing to be regulated.

## 7. Conclusions

The tests carried out for three different types of bed storage filling showed that the size of granite that is used as a storage material has a big impact on the efficiency of the process. The authors used granite that was previously prepared in the form of spheres, which allowed its characteristic dimensions to be precisely determined. Due to the preparation of the experiment plan, the number of required measuring series was decreased, and it was shown that the efficiency of charging the bed storage filled with granite is similar for all of the sizes of the filling material after the first hour of charging.

However, differences in the efficiency are visible later, and therefore, the time at which the research is conducted is important. As can be seen in Figure 9, Figure 10 and Figure 11, in the second and third hour, the efficiency of the charging process is much more dependent on the air inlet temperature and the flow rate of the working medium than in the first hour. Conducting research on the process of discharging the bed storage showed that it is possible to obtain similar heat fluxes as during the charging process under the same process conditions. Therefore, the authors believe that they showed that granite, due to its thermal and mechanical parameters, can be used as a filling of bed storage, as well as in solar dryers with a working medium in the form of air.

## Figures and Tables

**Table 1:** Assumed values of input factors.

Variable	−1	0	+1
*T_in_*, °C	90	120	150
${\dot{V}}_{a}$, m^3^/s	0.0065	0.0085	0.0105
*D*, mm	50	70	90

**Table 2:** Hartley design matrix for the experimental tests.

No.	*T_in_*	$\;{\dot{V}}_{a}$	*D*
	°C	m^3^/s	mm
1	150	0.0105	90
2	150	0.0065	50
3	90	0.0105	50
4	90	0.0065	90
5	150	0.0085	70
6	90	0.0085	70
7	120	0.0105	70
8	120	0.0065	70
9	120	0.0085	90
10	120	0.0085	50
11	120	0.0085	70

**Table 3:** The values used to determine heat losses from the dryer.

${\dot{V}}_{a}$	$T_{in.loss}\;$	$T_{out.loss}$	ΔT	$T_{avg}$
m^3^/s	°C	°C	°C	°C
0.0065	30.0	28.2	1.8	29.1
	90.0	78.3	11.7	84.2
	150.0	128.9	21.1	139.5
0.0085	30.0	29.0	1.0	29.5
	90.0	80.2	9.8	85.1
	150.0	131.9	18.1	141.0
0.0105	30.0	29.6	0.4	29.8
	90.0	81.7	8.3	85.9
	150.0	133.8	16.2	141.9

**Table 4:** The measured values and calculation results after three hours of charging.

Series No.	$\;{\dot{V}}_{a}$	$\;T_{in}$	$\;T_{out}$	$\;{\dot{Q}}_{act}$	$\;{\dot{Q}}_{max}$	ε
	m^3^/s	°C	°C	kW	kW	-
1	0.0105	150.0	122.7	0.111	0.252	0.440
2	0.0065	150.0	115.4	0.092	0.206	0.445
3	0.0105	90.0	79.0	0.040	0.124	0.325
4	0.0065	90.0	67.6	0.074	0.144	0.517
5	0.0085	150.0	115.8	0.122	0.248	0.494
6	0.0085	90.0	73.3	0.055	0.132	0.414
7	0.0105	120.0	98.0	0.088	0.200	0.441
8	0.0065	120.0	89.2	0.100	0.192	0.520
9	0.0085	120.0	95.7	0.093	0.198	0.470
10	0.0085	120.0	99.4	0.053	0.159	0.334
11	0.0085	120.0	94.0	0.084	0.186	0.450

**Table 5:** Measured values and calculations for experiment No. 9.

Time	${\dot{V}}_{a}$	$\;T_{in}$	$\;T_{out}$	$\;{\dot{Q}}_{act}$	$\;{\dot{Q}}_{max}$	ε
min.	m^3^/s	°C	°C	kW	kW	-
10	0.0085	120	47.9	0.520	0.600	0.866
20	0.0085	120	55.2	0.454	0.539	0.843
30	0.0085	120	60.9	0.398	0.486	0.820
45	0.0085	120	68.0	0.340	0.432	0.788
60	0.0085	120	74.3	0.293	0.388	0.754
75	0.0085	120	77.9	0.234	0.329	0.711
90	0.0085	120	82.3	0.200	0.298	0.671
120	0.0085	120	88.3	0.151	0.252	0.599
150	0.0085	120	92.1	0.116	0.219	0.531
180	0.0085	120	95.4	0.093	0.197	0.470

**Table 6:** Values of regression coefficients after one, two, and three hours of the charging process.

Series No.	Regression Coefficient Value After 1 Hour	Regression Coefficient Value After 2 Hours	Regression Coefficient Value After 3 Hours
1	−2.64223320 × 10^−1^	−2.07018750	−3.72080383 × 10^−1^
2	7.22428567 × 10^−3^	1.79728125 × 10^−2^	1.87816982 × 10^−3^
3	−7.39681743 × 10^−6^	−1.30555556 × 10^−5^	4.50243534 × 10^−6^
4	1.19773576 × 10^1^	4.18321875 × 10^1^	2.39270715 × 10^1^
5	−2.40393090 × 10^1^	−1.08625000 × 10^2^	−1.19461952 × 10^2^
6	1.03480696 × 10^2^	2.18868750 × 10^2^	−9.31543405 × 10^1^
7	3.44957618 × 10^2^	6.62500000 × 10^2^	7.77406397 × 10^3^
8	−5.16405955 × 10^−2^	−1.83822917 × 10^−1^	2.04561599 × 10^−2^
9	−6.70653395 × 10^−1^	−1.70145833	−6.15478181 × 10^−2^
10	−1.17647086 × 10^3^	−3.09437500 × 10^3^	−2.24288759 × 10^2^
11	6.66838861	2.17291667 × 10^1^	−4.25116346

## Nomenclature

$b_{i}$—*i*th regression coefficient, -
$c_{pa}$—air specific heat at constant pressure, kJ/kg∙K
*D*—diameter of granite spheres, m
${\dot{m}}_{a}$—air mass flow rate, kg/s
${\dot{Q}}_{act}$—actual heat flux of stored energy, kW
${\dot{Q}}_{max}$—maximum heat flux of stored energy, kW
${\dot{Q}}_{loss}$—heat loss, kW
$T_{in.loss}$—temperature at the inlet to the packed bed not filled with granite, °C
$T_{out.loss}$—temperature at the outlet to the packed bed not filled with granite, °C
$T_{in}$—air temperature at the inlet to the packed bed, °C
$T_{out}$—air temperature at the outlet of the packed bed, °C
${\dot{V}}_{a}$—air volume flow rate, m^3^/s
*x*—input parameter, -
ε—efficiency of the packed bed charging process, -
$\varepsilon_{t}\;$—theoretical efficiency of the packed bed charging process, -
$\rho_{a}$—air density, kg/m^3^

## References

[1] Sharma A., Chen C.R., Lan N.V. (2009). Solar-energy drying systems: A review. Renew. Sust. Energ. Rev..

[2] VijayaVenkataRaman S., Iniyan S., Goic R. (2012). A review of solar drying technologies. Renew. Sust. Energ. Rev..

[3] Fudholi A., Sopian K., Ruslan M.H., Alghoul M.A., Sulaiman M.Y. (2010). Review of solar dryers for agricultural and marine products. Renew. Sust. Energ. Rev..

[4] El-Sebaii A.A., Shalaby S.M. (2012). Solar drying of agricultural products: A review. Renew. Sust. Energ. Rev..

[5] Agrawal A., Sarviya R.M. (2016). A review of research and development, work on solar dryers with heat storage. Int. J. Sust. Energy.

[6] Bal L.M., Satya S., Naik S.N. (2010). Solar dryer with thermal energy storage systems for drying agricultural food products: A review. Renew. Sust. Energ. Rev..

[7] Kant K., Shukla A.A., Sharma A., Kumar A.J. (2016). Thermal energy storage based solar drying systems: A review. Innov. Food Sci. Emerg. Technol..

[8] Rabha D.K., Muthukumar P. (2017). Performance studies on a forced convection solar dryer integrated with a paraffin wax–based latent heat storage system. Sol. Energy.

[9] Reyes A., Mahn A., Vásquez F. (2014). Mushrooms dehydration in a hybrid-solar dryer, using a phase change material. Energ. Convers. Manag..

[10] Bhardwaj A.K., Chauhan R., Kumar R., Sethi M., Rana A. (2017). Experimental investigation of an indirect solar dryer integrated with phase change material for drying valeriana jatamansi (medicinal herb). Case Stud. Therm. Eng..

[11] Jain D., Tewari P. (2015). Performance of indirect through pass natural convective solar crop dryer with phase change thermal energy storage. Renew. Energ..

[12] Potdukhe P.A., Thombre S.B. (2008). Development of a new type of solar dryer: Its mathematical modeling and experimental evaluation. Int. J. Energy Res..

[13] Ayyappan S., Mayilsamy K., Sreenarayanan V.V. (2015). Performance improvement studies in a solar greenhouse dryer using sensible heat storage materials. Heat Mass Transfer.

[14] Mohanraj M., Chandrasekar P. (2009). Performance of a forced convection solar dryer integrated with gravel as heat storage material for chili drying. J. Eng. Sci. Technol..

[15] Jain D., Jain R.K. (2004). Performance evaluation of an inclined multi-pass solar air heater with in-built thermal storage on deep-bed drying application. J. Food Eng..

[16] Jain D. (2005). Modeling the system performance of multi-tray crop drying using an inclined multi-pass solar air heater with in-built thermal storage. J. Food Eng..

[17] Jain D. (2007). Modeling the performance of the reversed absorber with packed bed thermal storage natural convection solar crop dryer. J. Food Eng..

[18] Kozłowski S. (Poland 1986). Surowce Skalne Polski (Polish Rock Materials).

[19] Osiecka E. (2010). Materiały Budowlane, Kamień, Ceramika, Szkło (Building Materials. Stone, Ceramics, Glass).

[20] Nedelec A., Bouchez J.L. (2015). Granites, Petrology, Structure, Geological Setting, and Metallogeny.

[21] Amin M.N., Khan K., Saleem M.U., Khurram N., Niazi M.U.K. (2017). Aging and Curing Temperature Effects on Compressive Strength of Mortar Containing Lime Stone Quarry Dust and Industrial Granite Sludge. Materials.

[22] Freire-Lista D.M., Fort R., Varas-Muriel M.J. (2016). Thermal stress-induced microcracking in building granite. Eng. Geol..

[23] Cowie S., Walton G. (2018). The effect of mineralogical parameters on the mechanical properties of granitic rocks. Eng. Geol..

[24] Zhou J., Zhang L., Yang D., Braun A., Han Z. (2017). Investigation of the Quasi-Brittle Failure of Alashan, Granite Viewed from Laboratory Experiments and Grain-Based Discrete Element Modeling. Materials.

[25] Fan X., Lin H., Cao R. (2018). Bending Properties of Granite Beams with Various Section-Sizes in Three-Point Bending Tests. Geotech. Geol. Eng..

[26] Bai B., He Y., Li X., Li J., Huang X., Zhu J. (2017). Experimental and analytical study of the overall heat transfer coefficient of water flowing through a single fracture in a granite core. Appl. Therm. Eng..

[27] Kumari W.G.P., Ranjith P.G., Perera M.S.A., Chen B.K., Abdulagatov I.M. (2017). Temperature-dependent mechanical behaviour of Australian Strathbogie granite with different cooling treatments. Eng. Geol..

[28] Zeng Y., Tang L., Wu N., Cao Y. (2018). Numerical simulation of electricity generation potential from fractured granite reservoir using the MINC method at the Yangbajing geothermal field. Geothermics.

[29] Li B., Ju F. (2018). Thermal stability of granite for high temperature thermal energy storage in concentrating solar power plants. Appl. Therm. Eng..

[30] Nemś M., Kasperski J., Nemś A., Bać A. (2018). Validation of a new concept of a solar air heating system with a long-term granite storage bed for a single-family house. Appl. Energy.

[31] Cheng C., Li X., Li S., Zheng B. (2017). Failure Behavior of Granite Affected by Confinement and Water Pressure and Its Influence on the Seepage Behavior by Laboratory Experiments. Materials.

[32] Kolomá K., Červinka R., Hanusová I. (2018). ^137^Cs transport in crushed granitic rock: The effect of bentonite colloids. Appl. Geochem..

[33] Kasperski J., Nemś M. (2013). Investigation of thermo-hydraulic performance of concentrated solar air-heater with internal multiple-fin array. Appl. Therm. Eng..

[34] Nemś M., Kasperski J. (2016). Experimental investigation of concentrated solar air-heater with internal multiple-fin array. Renew. Energy.

[35] Wiset L., Srzednicki G., Driscoll R., Nimmuntavin C., Siwapornrak P. (2001). Effects of High Temperature Drying on Rice Quality. CIGR J. Sci. Res. Dev..

[36] Nemś M., Nemś A., Kasperski J., Pomorski M. (2017). Thermo-hydraulic analysis of heat storage filled with the ceramic bricks dedicated to the solar air heating system. Materials.

[37] Domański R. (1990). Magazynowanie Energii Cieplnej [Heat Energy Storage].

[38] Hartley H. (1959). Smallest composite designs for quadratic response surface. Biometrics.

[39] Jurlewicz T., Skoczylas Z. (2001). lgebra liniowa 1 [Linear Algebra 1].

[40] Singh R., Saini R.P., Saini J.S. (2006). Nusselt number and friction factor correlations for packed bed solar energy storage system having large sized elements of different shapes. Sol. Energy.

[41] Hossain M.Z., Hossain M.A., Awal M.A., Alam M.M., Rabbani A.H.M.M. (2015). Design and Development of Solar Dryer for Chilli Drying. Int. J. Res..

